# Analysing the impact of trade agreements on national food environments: the case of Vanuatu

**DOI:** 10.1186/s12992-021-00748-7

**Published:** 2021-09-16

**Authors:** Amerita Ravuvu, Joe Pakoa Lui, Adolphe Bani, Anna Wells Tavoa, Raymond Vuti, Si Thu Win Tin

**Affiliations:** 1 Non-Communicable Disease Policy & Planning Adviser, Public Health Division, Pacific Community (SPC), Private Mail Bag, Suva, Fiji; 2 Department of External Trade, Ministry of Foreign Affairs, International Cooperation and External Trade, Government of the Republic of Vanuatu, Port Vila, Vanuatu; 3 Department of External Trade, Trade Negotiation Division, Ministry of Foreign Affairs, International Cooperation and External Trade, Government of the Republic of Vanuatu, Port Vila, Vanuatu; 4 International Trade Merchandise Statistics, Vanuatu Statistics Office, Ministry of Finance and Economic Management, Government of the Republic of Vanuatu, Port Vila, Vanuatu; 5 Vanuatu Investment Promotion Authority, Port Vila, Vanuatu; 6 Non-Communicable Diseases, Public Health Division, Pacific Community (SPC), Suva, Fiji

**Keywords:** Trade, Trade agreements, Vanuatu, Food imports, Foreign investment, Non-communicable diseases, Monitoring

## Abstract

**Background:**

A large body of literature exists on trade liberalisation and the ways in which trade agreements can affect food systems. However, the systematic and objective monitoring of these and their impact on national food environments has been limited. Using a case study, this paper undertakes a systematic analysis of how Vanuatu’s obligations under WTO agreements has impacted its food environment.

**Results:**

Data collection was guided by the INFORMAS trade monitoring framework’s minimal approach and seven selected indicators outlined in three domains: trade in goods, trade in services and FDI, and policy space. Strong associations between trade liberalisation and imported foods, especially ultra-processed foods were evident in measured indicators as follows: (i) food trade with 32 WTO countries showing high levels of import volumes; (ii) a marked increase in ‘less healthy’ focus food imports namely fatty and other selected meat products, sugar, savoury snacks, ice-cream and edible ices and energy-dense beverages; (iii) actual and bound tariff rates impacting import trends of ice-cream and edible ices, bakery products and confectionary; and in other instances, a sharp increase in import of crisps, snacks and noodles despite tariff rates remaining unchanged from 2008 to 2019; (iv) policies regulating food marketing, composition, labelling and trade in the domestic space with relatively limited safeguard measures; (v) 49 foreign-owned food-related companies involved in food manufacturing and processing and the production of coffee, bakery products, confectionary, food preservatives, fish, local food products and meat, and the manufacturing, processing and packaging of palm oil, coconut oil, cooking oil, water, cordial juice, flavoured juices, soft drinks and alcoholic beverages. These were largely produced for local consumption; (vi) 32 domestic industries engaged in food and beverage production; and (vii) an assessment of WTO provisions relating to domestic policy space and governance showing that the current legal and regulatory environment for food in Vanuatu remains fragmented.

**Conclusions:**

The analysis presented in this paper suggest that Vanuatu’s commitments to WTO agreements do play an important role in shaping their food environment and the availability, nutritional quality, and accessibility of foods.

**Supplementary Information:**

The online version contains supplementary material available at 10.1186/s12992-021-00748-7.

## Background

Food environments globally are dominated by highly accessible and heavily promoted ultra-processed foods [1–3]. Food environments – the physical, economic, political and socio-cultural contexts in which people engage with the food system to make decisions about food acquisition, preparation and consumption within the wider food system [4] are inextricably linked to noncommunicable diseases (NCDs) [5]. The rapid rise in NCDs globally and in the Pacific region in recent decades poses an urgent and serious health and development challenge. Dietary-risk factors are a major driver of preventable deaths due to overweight, obesity and diet-related NCD globally [6, 7]. In the Pacific region, which has been called the NCD capital of the world, approximately 75% of death that are largely premature are due to NCDs [8].

A wide range of policies shape food environments, including government policies covering food composition, labelling, marketing, availability and price, across a broad range of sectors: health, agriculture, education and trade. Trade policies regarding liberalisation, export promotion, import substitute measures, protection of domestic industries and support for foreign direct investment have also contributed to the increased availability of foods associated with the nutrition transition. Throughout the Pacific region, there is some evidence of a correlation between the introduction of free trade/trade agreements and the increasing availability/consumption of ultra-processed foods high in trans-fat, sugar and salt [9–11]. Three key trade-related changes contributing to this are: (i) the opening of domestic markets to international food trade; (ii) increased entry of transnational food companies and greater foreign direct investment; and (iii) intensified global food marketing and promotion [12–14].

Vanuatu is an archipelago nation in the Melanesian group of the Pacific region with a population of about 300,000 people. Its economy relies primarily on agriculture, with the key crops of kava, copra, coffee and cocoa [15]. Despite its rich and lush vegetation with a wide range of fresh fruits and vegetables grown locally, Vanuatu is also a food import dependent nation with an increasing presence of ultra-processed foods [16]. Vanuatu joined the World Trade Organisation (WTO) in 2012 as an LDC (least-developed country) and has only recently graduated to its low- and middle-income country (LMIC) status in December 2020. However, when this study was undertaken Vanuatu was still recognised as an LDC. While this study focuses on a review of Vanuatu’s commitments under WTO trade agreements only, Vanuatu is also party to several other agreements including the Melanesian Spearhead Group Trade Agreement (MSGTA) which it joined in 2005, the Pacific Agreement on Closer Economic Relations (PACER-Plus) which it joined in 2017 and the Pacific Island Countries’ Trade Agreement (PICTA) which it joined in 2001. Due to reduced technical barriers to trade, these trade agreements will all have implications for the availability of ultra-processed foods imported into Vanuatu, making it even more pertinent the need to assess the implications for policy space in Vanuatu to enable it to protect its food environment against these harmful commodities [14, 17].

The Pacific NCD roadmap, developed at the request of Pacific finance and economic ministers in 2014, recommended five key strategies for adoption by Pacific countries in their own NCD country roadmaps to intensify response to the Pacific NCD crisis. In line with the Pacific NCD roadmap [18], the *Vanuatu NCD Roadmap 2015–2018* calls for national action to reduce premature NCD mortality by 25% by 2025. The four major NCDs, namely cardiovascular disease, diabetes, cancers and chronic respiratory diseases, are now responsible for approximately 60% of all premature deaths in Vanuatu [19]. Vanuatu’s mortality target is also in line with the Sustainable Development Goal (SDG) target 3.4 calling for a reduction of premature NCD mortality by a third by 2030 relative to 2015 levels [6, 20] and the World Health Organization NCD global mortality target comprising of a 25% reduction in premature mortality from NCDs by 2025 [21]. The presence of these largely preventable diseases and the magnitude of this disease burden has widespread implications for individuals, families and communities in Vanuatu. Of major concern is the excessive consumption of salt and increased consumption of high fat and trans-fat foods as key contributors to cardiovascular diseases and diabetes.

Despite Vanuatu’s accession to the WTO, and its high burden of obesity and diet-related NCDs, there has been no systematic assessment and monitoring of its food environment and their relationship with Vanuatu’s NCD rates. To address this gap, the aim of this study is to establish baseline information on the volume and types of unhealthy imported foods entering Vanuatu as a result of its WTO trade commitments. This will better inform and guide trade policies and legislations that promote healthy diets.

## Methods

Given the focus of this study on the links between trade and food availability at the national level, the INFORMAS trade monitoring framework and its associated data collection protocol (see Table 1) were used to guide the selection of indicators and analysis [14]. The first step involved a desktop review to map and record Vanuatu’s existing food and trade-related policies, as well as its commitments under the WTO (World Trade Organization) trade agreements that have implications for Vanuatu’s national food environment, under three domains of the INFORMAS framework: (i) trade in goods; (ii) trade in services and foreign direct investment; and (iii) policy space.

**Table 1:** INFORMAS Trade Monitoring Framework. Suggested step-wise framework for monitoring the impacts of trade agreements on national food environments.

***Domain:***	***‘Minimal’ approach:***
1. Trade in goods	• Provisions in text relating to tariff and non-tariff barriers to trade, including tariff-rate quotas, import licensing and price-banding) and specific food categories affected by these provision • Total food import volumes • Focus food category import volumes • Rate of change in total food import volumes • Rate of change in focus food category import volumes • Actual and bound tariff rates for focus food categories • Tariff-rate quotas for focus food categories •Tariff differential (if any) between healthy and unhealthy focus food categories
2. Trade in services & FDI	• Provisions in text relating to restrictions on foreign ownership, intellectual property (IP) protection, performance requirements for foreign investors, and national treatment • Type and country of origin of all foreign-owned TFCs operating in country • FDI investment in food production, processing, retail and advertising sectors (monetary value) • Rate of change in total inward FDI in food and related sectors (including communications and advertising)
3. Domestic protections & supports	• Provisions in text relating to domestic protections and supports such as agricultural safeguards, special treatment of agricultural products, anti-dumping and countervailing measures, agricultural supports and export subsidies and promotion
4. Policy space & governance	• Provisions in text relating to domestic policy space and governance (including government procurement, enforcement, transparency, dispute settlement and government regulation of food marketing, composition, labelling)

### Selection of focus foods

Within the ‘minimal’ version of the INFORMAS trade monitoring framework, a set of focus foods rather than the total food supply was selected. These foods were identified and classified as ‘healthy’ or ‘less healthy’ and these are based on the suggested focus food categories identified in Box 3 of the INFORMAS trade monitoring framework paper [14] and reflected in Table 2 and Table 5. Specific food categories were selected based on Vanuatu data captured in a shop survey conducted in 2017 [22] and a baseline assessment identifying food items most important to nutrition in Vanuatu [23].

**Table 2:** Less healthy focus foods selected with their corresponding HS Code

Unhealthy Focus Food Category	Food sub-category	HS Code	Product
Edible oils and spreads	Cooking Oil	151,110, 151,190 151,321, 151,329 15,162,000, 15,119,000	Palm Oil
		15,152,100, 15,152,900 15,152,100, 15,159,000	Corn Oil
	Edible Oil	1,501,000, 15,019,000 1,502,000, 1,503,000 15,162,000, 15,179,010, 15,179,090, 15,179,000 15,180,000, 15,220,000	Hydrogenated fats, lard, dripping
	Spread	15,171,000, 15,179,000	Margarine
		0403900, 04041000 04051000, 04052000 04059000, 15,171,000 15,179,000, 1,804,000 20,071,000, 20,079,900 20,081,100, 20,081,900 20,089,900, 20,091,900 21,069,000, 22,087,010	Butter, Peanut butter
Fatty meat products	Processed meats	02031200, 02032200 02032900, 02071490 02089000, 02101100 02101200, 02101900 02109900, 16,010,000 16,021,000, 16,022,000 16,023,100, 16,023,200 16,023,900, 16,024,100 16,024,900, 16,025,000 16,025,090, 16,029,000 16,029,020, 16,029,030 16,029,090	Sausage, ham, bacon, salami, jerky, cold cuts, chicken nuggets, patties
	Canned meat	16,010,000, 16,021,000 16,022,000, 16,023,100 16,023,200, 16,023,900 16,024,100, 16,024,900 16,025,010, 16,025,090 16,029,010, 16,029,020 16,029,030, 16,029,090	Corned mutton, corned beef, spam, canned chicken, ham, turkey, etc.
High-fat/processed dairy products	Cheese	04063010, 04062000 04069000	Processed cheese
	Yoghurt	04031090, 4,039,000	Fruit-based/Flavoured
	Ice-cream and edible ices	21,050,000, 21,069,000	Ice-cream and edible ices
Energy-dense beverages	Cordial	20,091,200, 20,091,900 20,093,900, 20,097,900 20,098,100, 20,098,900 20,099,000, 21,069,000 22,029,000	Cordial/Concentrate/Powder
	Soft drink	22,019,000, 22,021,000 22,029,000, 22,029,100 22,029,900	Sugar-sweetened
	Electrolyte drinks	22,019,000, 22,021,000 20,091,200, 20,098,900 22,029,900	Sports drinks
Sugar and other caloric sweeteners	Sugar	17,011,100, 17,011,200 17,011,300, 17,011,400 17,019,100, 17,019,900 17,021,100, 17,021,900 17,022,000, 17,024,000 17,026,000, 17,029,000	Natural cane and refined sugar
Savoury ready to eat snacks	Crisps and snacks	19,030,000, 19,041,000 19,042,000, 19,049,000	Snack packs, corn chips, potato chips, other (Dried peas etc.)
	Noodles	19,021,900, 19,022,000 19,023,000, 19,041,000 19,049,000	Instant, Flavoured
Sweet snacks	Confectionary	17,041,000, 17,049,000 1,801,000, 18,031,000 18,040,000, 18,050,000 18,061,000, 18,062,000 18,062,010, 18,063,100 18,063,200, 18,069,000 18,069,090, 19,041,000 19,049,000	Chocolate and sweets (Chocolate based; sugar based) Chewing gum
	Bakery Products	18,062,000, 18,069,000 1,905,100, 1,905,200 19,053,000, 19,053,100 19,053,200, 19,054,000 19,059,000	Sweet biscuits Cakes and pastries

### Defining the categories

The food and drink categories listed in Table 2 are typically ultra-processed except for vegetable/cooking oils and yet provide little or no nutritional benefit that is required for a healthy diet. These foods are classified and reported in this assessment as ‘less healthy’ focus foods that are frequently consumed and have a significant negative impact on diet quality. They are: edible oil and spreads (including hydrogenated oils used as an ingredient in processed foods); fatty meat products (e.g. turkey tails, mutton-flaps, processed meats); high fat processed dairy products (e.g. processed cheese, ice cream); energy-dense beverages (e.g. carbonated soft drinks); sugars and other caloric sweeteners (including High-Fructose Corn Syrups); savoury ready-to-eat snacks and meals (e.g. potato chips, French fries, instant noodles); and sweet snacks (e.g. biscuits, pastries, confectionary). Table 2 summarises the focus foods and Harmonized System (HS) Codes capturing these food products with the data provided by the Vanuatu National Statistics Office (VNSO).

### Food-related trade indicators

Drawing on the four domains of the INFORMAS monitoring framework, in this analysis, the focus was on the minimal monitoring approach in three of the four domains: trade in goods, trade in services and foreign direct investment, and policy space (see Table 1). In terms of domestic protections and supports, Vanuatu has no programmes or policies that are subject to reduction commitments within the meaning of Article 6 of the WTO Agreement on Agriculture, so this has been excluded. Data were obtained for the following indicators: (i) total food import volume with WTO member countries; (ii)‘less healthy’ focus food category import volumes; (iii) actual and bound tariff rates for the ‘less healthy’ focus food category; (iv) the type and country of foreign-owned food and beverage industries operating in Vanuatu and the monetary value of their foreign direct investment; (v) the type of domestic industries engaged in the food and beverage sector; and (vi) the provisions in WTO trade agreements relating to domestic policy space and governance. For total food import volumes, data were selected by food import categories as defined by the VNSO and the specific Harmonized System (HS) classification codes used to classify these food items. The selection of ‘less healthy’ foods to monitor for Vanuatu was based on the shop survey and consultation with Vanuatu Ministry of Health officials. Actual and bound tariff rates were provided by the Vanuatu Customs and Inland Revenue Department. Information about each trade policy and agreement was collected from various sources. The trade policies and legislations were accessed from various government ministry websites. Information on foreign direct investment and domestic industries was supplied by the Vanuatu Investment Promotion Authority and the Ministry of Tourism, Trade, Industry, Commerce and Ni-Vanuatu Business respectively. The food and agriculture related WTO trade agreements were collected from the WTO online database (www.wto.org). The scope of the review of the WTO agreements identified general rules that apply to Vanuatu as an LDC (least developed country) member of WTO, and specific commitments were listed as ‘schedules of specific commitments’. These reflect specific tariff concessions for the goods schedule (General Agreement on Tariffs and Trade – GATT), the specified level of market access and national treatment for the services schedule (General Agreement on Trade in Services – GATS), and specific services commitments that Vanuatu has given in the context of trade negotiations. Data on these indicators were analysed using Microsoft Excel and compared with changes in Vanuatu’s WTO commitments and domestic regulations in order to shed more light on the impact of trade. There were 32 **major** WTO trading partner countries exporting food into Vanuatu in 2019. Details of ‘other countries’ trading with Vanuatu were not segregated, so only major partners are reflected. The decision regarding the years to monitor food import volumes was based on the escalation of imports prior to Vanuatu joining WTO in 2012 and the trend of import volumes after the ratification and sign-off on WTO agreements and their implementation phases. Detailed food import volume data were collected from VNSO’s Automated System of Customs Data, which captures and implements all international standards for trade data by specific HS Code categories.

## Results

Trade agreements are an upstream determinant of population health [24–27] and Friel et al., have shown that trade in goods, trade in services and policy space influence the liberalization of the food trade which consequently results in increased food imports [14]. Drawing on Vanuatu’s trade in goods commitments, total food import data is presented to show how trade liberalisation has increased importation of foods which can impact consumption. It can also lead domestic firms in Vanuatu to lower prices if there is increased competition from international imports [26]. Less-healthy food data is also presented to illustrate how Vanuatu’s food trade has resulted in an increase in ultra-processed foods. Drawing on Vanuatu’s trade in services commitments, data showing the scope and scale of production through FDI and subsequent competition with domestic firms is also presented. This is to illustrate how changes to production can affect consumption of food and beverages via increased FDI in domestic production and intensified local competition [26].

To achieve a healthy food system, regulation across the supply chain is required but binding trade agreements may be constraining policy space for regulatory intervention in a way that hinders the uptake of effective nutrition policy [17]. Vanuatu’s WTO commitments and accession package outlined in this section clarify important considerations that can have implications for the design of public health nutrition regulations or policy and Vanuatu’s food environment more broadly in relation to international trade law, and in particular, to the principles of non-discrimination, transparency, necessity and justification, dispute processes, harmonisation, market access requirements and quantitative restrictions affecting trade in goods (GATT – General Agreements on Tariffs and Trade) – with food being one of the most highly traded goods globally, and trade in services (GATS – General Agreement on Trade in Services).

### Total food import volumes

Increased trade liberalisation and the liberalization of food trade results in increased food imports, both healthy and unhealthy, although there is more of the latter with an increase in imported animal products and ultra-processed food [14]. Consequently, an increase in the latter also increases its consumption which ultimately leads to poor health outcomes [2].

**Fig. 1:**
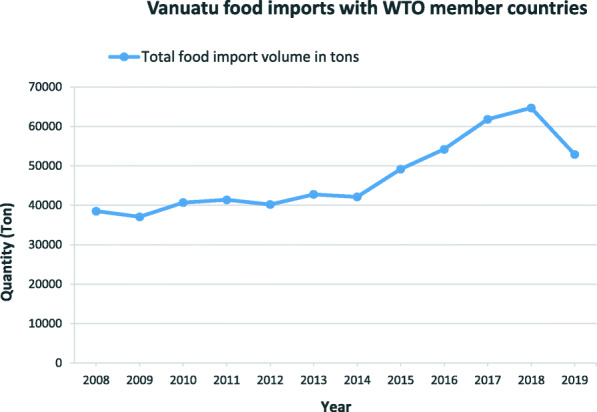
Vanuatu total import volume with 32 WTO member countries for the period between 2008 to 2019. *Source:* Vanuatu National Statistics Office

Data on the total volume of food imports into Vanuatu were collected from the 32 major WTO trading partners. The data include volumes of animal products, vegetable products, prepared foodstuffs, miscellaneous food preparations, non-alcoholic beverages, and animal or vegetable oils and fats (HS 01–2501). These food categories excluded variations of products used for pharmaceuticals, animal feeds, live animals, and flower cuttings and seeds not listed as edible. As Fig. 1 shows, there were high and increasing levels of food import volumes from these WTO member countries between 2008 and 2019, a slight decline in 2009, 2012 and 2014, and a sharp increase from 2015 to 2018. In 2019, there was a sharp decline.

### Less healthy food categories import volumes

**Fig. 2:**
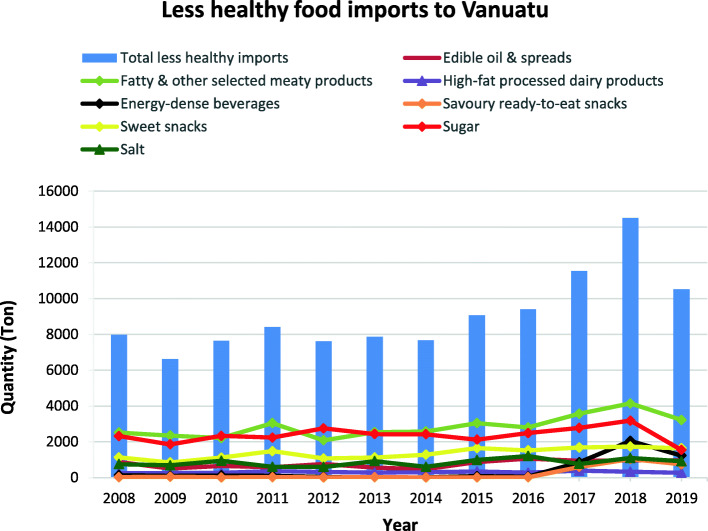
Volume of select less healthy food imports to Vanuatu over the period between 2008 to 2019 from major WTO importing countries. *Source:* Vanuatu National Statistics Office

**Fig. 3:**
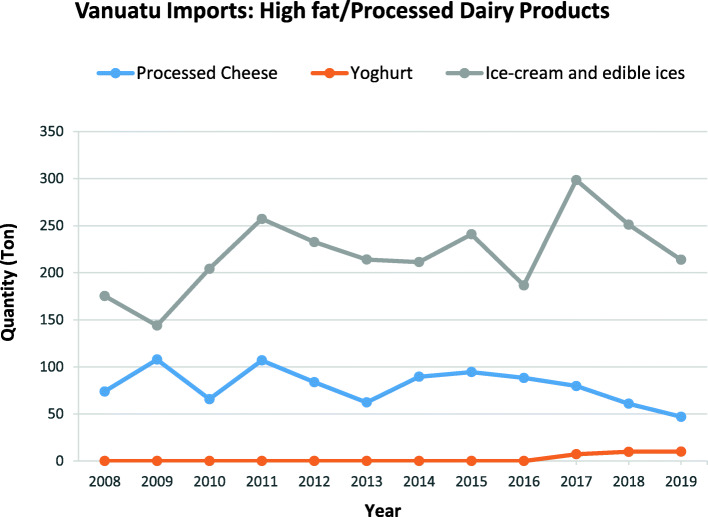
High fat/processed dairy food imports to Vanuatu over the period between 2008 to 2019 from major WTO importing countries. *Source:* Vanuatu National Statistics Office

**Fig. 4:**
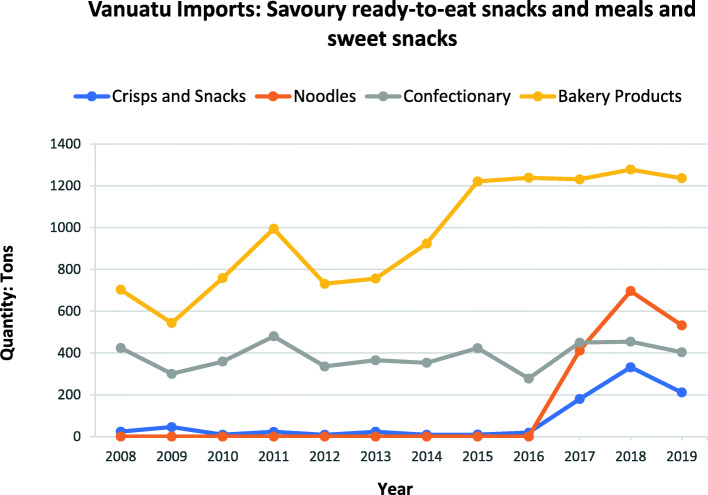
Savoury ready-to-eat snacks and meal (crisps and snacks, noodles) and sweet snack (bakery products, confectionary) imports to Vanuatu over the period between 2008 to 2019 from major WTO importing countries. *Source:* Vanuatu National Statistics Office

**Fig. 5:**
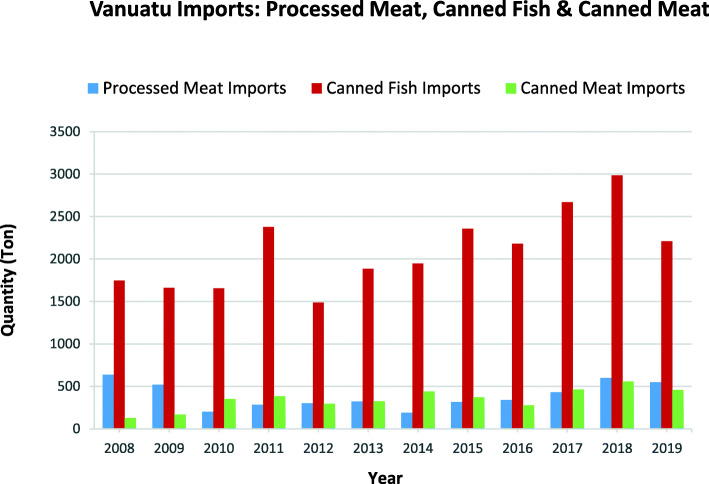
Selected fatty and other meat imports into Vanuatu over the period between 2008 to 2019. *Source:* Vanuatu National Statistics Office

**Fig. 6:**
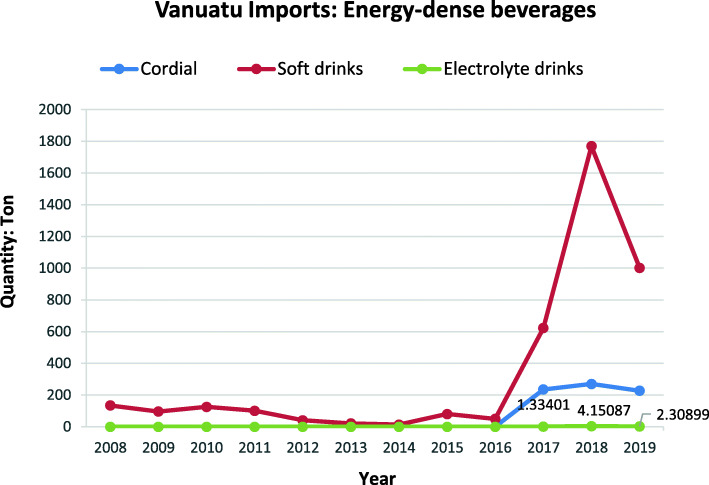
Cordial juices, soft drinks and energy drink imports into Vanuatu over the period between 2008 to 2019. *Source:* Vanuatu National Statistics Office

**Fig. 7:**
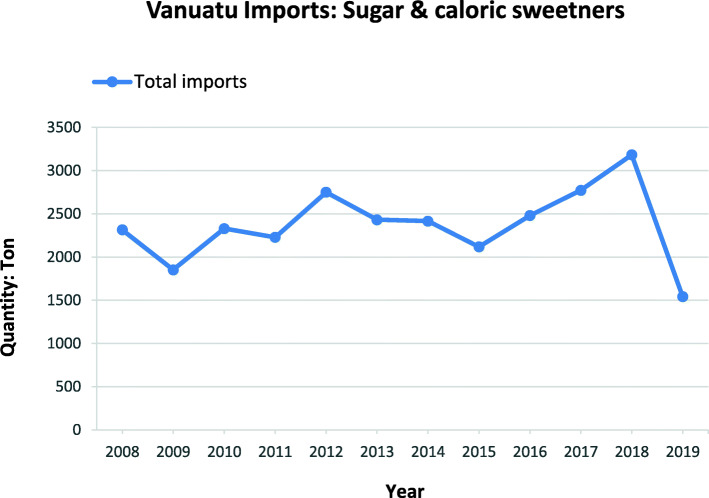
Sugar and caloric sweetener imports into Vanuatu over the period between 2008 to 2019. *Source:* Vanuatu National Statistics Office

Figures 2, 3, 4,5, 6 and 7 illustrate the changes in import volumes for the various ‘less healthy’ food categories from 2008 to 2019. Figure 2 shows a marked increase in fatty and other selected meat products, sugar, savoury ready-to-eat snacks and energy-dense beverages between 2016 and 2018.

Acceding LDCs are required to bind all agricultural tariff lines at an overall average rate of 50% and, in line with the WTO agreement on agriculture, all the members are required to bind all agricultural tariff lines. On accession to WTO in 2012, Vanuatu bound all its agricultural tariff lines (including food products) at an overall average rate of 43.6%. For non-agricultural products, the WTO decision provides two options: acceding LDCs shall bind 95% of their NAMA (non-agricultural market access) lines at an overall average rate of 35%, or they can undertake more comprehensive binding coverage. Vanuatu agreed to a 100% binding coverage of its NAMA tariff lines at an overall average rate of 39.1%. This decision to apply benchmark *ad valorem* rates to agricultural and NAMA tariff lines does not, however, prevent LDCs like Vanuatu from negotiating higher rates for sensitive lines, as it does not impose any tariff cap. For instance, Table 4 (see Additional file 1) shows that bound tariff rates for chicken, ice-cream and edible ices, cordials/juices, soft drinks and electrolyte drinks/sports drinks all have tariff peaks that exceed the benchmark. Table 4 also details the variations in tariff rates applied to the selected less healthy foods (shown in Fig. 2). Tariff rates for these foods remained the same, except for peanut butter, which had a 10% decrease in 2012 and then a 10% increase from 2017; fruit based/flavoured yoghurt, which had a 15% decrease from 2012; and margarine, which had a 10% decrease and tariff reduced to zero from 2017. Tariff rates for these categories are relatively low, compared to the rates for selected ‘healthy’ focus foods shown in Table 5 (see Additional file 2).

As part of its WTO obligation, Vanuatu grants MFN (most favoured nation) tariff treatment to all its trading partners. There appears to be sufficient policy space for protecting domestic sectors. Vanuatu also applies preferential tariffs to parties of the MSGTA, The Pacific Island Countries Trade Agreement (PICTA) and, upon ratification, the Pacific Agreement on Closer Economic Relations Plus (PACER Plus). In addition to these, there are two charges affecting food imports, as well as domestic products: VAT and excise duty. VAT of 15% applies to all goods and services unless they are exempt or zero-rated. Imports are VAT exempt if they are valued at VUV 10,000 or less. Until 2017, the VAT rate had been 12.5% but this was increased to support fiscal consolidation. An excise duty applies to items such as alcohol and tobacco products and it is now also applied to sugar-sweetened beverages. In 2015, a specific excise tax was applied to both imported and locally produced sweetened beverages (HS 2022). The tax rate is 50 vt/L, but for imported sweetened beverage products, there is an additional 75% tariff applied (see Table 4 in Additional File 1).

Tariff rates for ice-cream and edible ices, savoury ready-to-eat snacks (crisps and snacks, noodles) and sweet snacks (bakery products – sweet biscuits, confectionary) remained the same from 2008 to 2019 (see Table 4 in Additional File 1). Despite this, there have been increases in the import of ice cream and edible ices (See Fig. 3), while the import of bakery products and confectionary increased from 2009 to 2011 and there was a sharp increase in the import of crisps and snacks and noodles from 2016 to 2018 (see Fig. 4).

As shown in Fig. 5, from 2008 to 2019, Vanuatu consistently imported more canned fish than processed meat and canned meat. With applied tariffs on processed meat and canned meat imports remaining unchanged between 2008 and 2019 (with the exception of a 10% increase in the tariffs on imported ham, bacon, salami, jerky, cold cuts and chicken nuggets from 2012 to 2019), there have been no marked increases. An additional excise duty of 20% (from 2014) and VAT of 15% (from 2017) applies to these food categories.

Figure 6 shows an increase in soft drink and cordial imports from 2016 to 2018, despite a high applied tariff of 75% on soft drinks and the applied tariff on cordial imports remaining unchanged from 2008 to 2019 at 20% per cent. The 75% per cent tariff applied to soft drinks and electrolyte/sports drinks is in addition to the specific excise tax rate of 50 vt/L applied since 2015 as alluded to earlier.

Sugar and caloric sweetener imports (HS 1701 and 1702) into Vanuatu over the period between 2008 to 2019 was varied (see Fig. 7). While the applied tariff on sugar and caloric sweetener imports remained unchanged at 10% from 2008 to 2019, an additional excise duty of 20% (From 2014) and VAT of 15% (From 2017) applies to these HS category codes.

### Trade in services and foreign direct investment

Following its accession to WTO in 2012, Vanuatu agreed to undertake specific commitments on 10 service sectors and to progressively liberalise its business environment, with few restrictions on investment in order to promote small local businesses. In accordance with the GATT Article III on national treatment, and paragraphs 1 to 3 in Article XVII of GATS, Vanuatu has applied no limitations on market access and no limitations on national treatment for the foreign investors. Only normal government approval and registration are required for all foreign investors under Vanuatu’s Foreign Investment Act No.15 of 1998 and its amendments.

### Type and country of origin of foreign-owned industries operating in Vanuatu

Table 6 (see Additional file 3) shows foreign-owned transnational corporations engaged in food and beverage production in Vanuatu from 1998 to 2019. While there are no available FDI (foreign direct investment) data specific to their investment in domestic food production, processing, retail and advertising sectors, the available data show that there were 49 foreign-owned companies engaged in food production, processing, wholesale and retail in Vanuatuin 2019 (see Additional file 3). These companies are mainly associated with food manufacturing and processing and the production of coffee, bakery products, confectionary, food preservatives, fish, local food products and meat, as well as the manufacturing, processing and packaging of palm oil, coconut oil, cooking oil, water, cordial juice, flavoured juices, soft drinks and alcoholic beverages. The production of these is largely for local consumption. As indicated in Table 6 (see Additional file 3), there are several breweries/distilleries, bakeries, cafes, restaurants and takeaway services with significant investment that are also operating in-country.

### Type and country of domestic industries in the food and beverage sector in Vanuatu

Vanuatu’s domestic food and beverage industries also play an important role in shaping Vanuatu’s food environment. Data provided by the Ministry of Industry and Trade show 32 locally owned food and beverage companies registered since 2014 (see Table 7 in Additional file 4). Of the 32, five sell confectionary, ice cream and frozen dessert products; one is engaged in the production of peanut oil; one sells flavoured juices/soft drinks; five are breweries/distilleries/liquor businesses; three sell bakery products and ready meals; one sells sweet savoury snacks; and nine sell meats, of which one specifically sells canned meats only and eight sell fresh and processed meats. The remaining seven companies are engaged in other business ventures, producing and selling water (2), fruit juices and frozen fruit delights (1), coffee (1), frozen root crops (1), dried spices, fruits and vegetables (1), and manioc flour (1).

### Summary of WTO commitments and Vanuatu’s accession package

At the Eighth WTO Ministerial Conference in December 2011, trade ministers decided to “further strengthen, streamline, and operationalise the 2002 LDC accession guidelines,” with the inclusion of benchmarks on goods and services, as well as elements on special and differential treatments, transition periods, transparency, and technical assistance. Table 3 summarises Vanuatu’s commitments and accession package, the relevant trade and investment principles that can influence its policy space and anticipated impacts on its national food environment.

**Table 3:** Summary of WTO Commitments and Vanuatu’s accession package, the relevant trade and investment principles that can influence on Nutrition Policy Space and anticipated impacts on Vanuatu’s national food environment

Commitments under the WTO agreements	Relevant principles	Anticipated impacts on Vanuatu’s food environment
1. Not carry out pre-shipment inspection of imports with no plan to do so.	**Harmonisation and international standards** Found in: WTO: GATT, Agreement on Preshipment Inspection Article 2 & 3	Pre-shipment checks are considered a technical barrier to trade. Vanuatu does not carry out any preshipment inspection with no intention of doing so increasing the risk of receiving poor-quality food products that may hinder the enforcement of any food composition laws.
2. Not apply any anti-dumping, countervailing or safeguard measures until it had implemented appropriate laws consistent with WTO agreements.	**Harmonisation and international standards** Found in: WTO: GATT Article 6, Agreement on Subsidies & Countervailing Measures	With the intent to enable a government to act against dumping where it is hurting domestic industries or to cope with a sudden surge of foreign goods, Vanuatu agreed to not applying any of these measures. This increases the risk of low-priced food imports that may hinder the enforcement of domestic food regulations.
3. No intention of being part of the Government Procurement Agreement (GPA)	**Transparency/Notification** Found in: WTO: Government Procurement Agreement, TBT Article 2.9 GATS Article 3	While being first and foremost a trade instrument, the GPA is an important tool in addressing two key governance challenges: (i) ensuring integrity in the procurement process (preventing corruption on the part of public officials) and (ii) promoting effective competition among suppliers [28]. When neither of these are regulated, it can impact on public procurement processes including public food procurement (production, productivity, diversification, non-farm activities, social protection and nutrition-specific interventions) and targeted agricultural interventions. As such, public food procurement strategies to promote agricultural development and improve food security and nutrition may not offer optimal nutrition where pro-competitive, transparency-enhancing internal reforms are absent.
4. Submit all notifications required by any agreement	**Transparency/Notification** Found in: WTO: TBT Article 2.9 GATS Article 3	This is relevant and applicable to Vanuatu’s food environment. Any new or amended food rules/policy introduced will require appropriate notification to be given where there is a technical barrier to trade or services.
5. Apply an average final bound rate of 39.7% (43.6% for agricultural products and 39.1% for industrial products) and binding all of its tariffs.	**Harmonisation and international standards** Found in: WTO: TBT Article 2.4 Agreement on Agriculture	Tariffs can affect the flow of trade. While Vanuatu has agreed to bind all its tariffs, the relationship of tariffs on the influx of food imports is not straightforward. Vanuatu will need to consider other complementary actions, for instance, as a source of revenue generation, is revenue from tariffs on imported foods earmarked for public health promotion efforts.
6. Having no export subsidies applied to agricultural products from 2013 to 2019.	**Harmonisation and international standards** Found in: WTO: TBT Article 2.4 Agreement on Agriculture Article 8 and 9	For underdeveloped agricultural sectors like Vanuatu, moderate levels of border protection may be appropriate to promote productivity and provide the stability needed for producers to react positively to incentives. Export subsidies have been used to promote market access in import markets so Vanuatu’s commitment not to have any can safeguard against cheap food imports below production costs entering and undermining local producers.
7. Applying import duty exemptions for goods imported for agriculture, horticulture, livestock and forestry. These include plant machinery, materials, equipment, spare parts and accessories. In addition to this, agricultural incentives are offered to agricultural producers and aid-financed programmes of domestic support for agriculture within the *de minimis* ceiling of 10%, given Vanuatu’s LDC status.	**Non-discrimination** ***MFN and National Treatment*** Found in: WTO: GATT Art.1.1 (MFN), Art 3.2 & 3.4. General exception XX(b) and Chapeau; TBT Art.2.1; SPS Art 5.5 **Necessity** Found in: WTO: Gatt Gen. exception XX(b); TBT Art 2.2; GATS Art.14 **Harmonisation and international standards** Found in: WTO: TBT Article 2.4	As an incentive, import duty exemptions can have a critical role in kick-starting agricultural productivity improvements and plays a role more broadly in promoting better outcomes in most dimensions of food security. This augurs well for Vanuatu’s Ministry of agriculture, livestock, fisheries, forestry and biosecurity and supports efforts of growing local, eating local and safeguarding for food nutrition and security.
8. Undertake specific commitments on 10 service sectors^1^ and 72 sub-sectors	**Regulatory coherence** Found in: WTO: TBT Article 2.7, 2.9 & 2. 10 GATS	The encouragement of FDI increases the availability of processed and ultra-processed foods and ultimately the consumption of these.
9. Progressively liberalising its business environment with few restrictions on investment to promote small local businesses.	**Harmonisation and international standards** Found in: WTO: TBT Article 2.4 GATS **Regulatory coherence** **Fair and Equitable Treatment**	The encouragement of FDI to promote local businesses will contribute to an increase in food preparations via domestic industries and the increased consumption of ultra-processed foods produced locally.

^1^Business; communication; construction and related engineering; distribution; education; environment; finance; health and social (hospital services & social services); tourism and travel-related; and transport services

## Discussion

A variety of drivers and policies (or the lack thereof) influence food systems and shape food environments. Globally, food systems have been challenged by population growth, globalisation, urbanisation and climate change and have been altered by agricultural, economic, trade, environmental and international development policies. These create incentives and disincentives which ultimately determine the production of particular types of food. These coupled with rural development, urban planning and transport policies determine the affordability of food and what food reaches which consumers [5]. Consumers’ dietary behaviours are also affected by several other factors that range from the personal – such as culture, knowledge, skills, dietary preferences, and time for food preparation – to economic and political – such as the cost or availability of food. Information about food, whether through education or marketing also influences food choices. Marketing, labelling and policies that have an impact on price affect consumers. Marketing has become so extensive, and billions of dollars are spent annually marketing foods high in fat, sugar and salt. Food marketing to children are also widespread across all the world and most of the marketing targeted at children focus on foods with a high content of sugar, fat or salt [29]. Consequently, today’s food systems are flooded with ultra-processed foods that have become cheaper and more widely available [2, 30]. Diet-related NCDs remain a significant contributor to the global prevalence of adult obesity which has nearly tripled since 1975, and a ten-fold increase of childhood overweight and obesity over the same period [31].

To better understand the links and impacts of trade on Vanuatu’s food environment, the baseline results presented in this paper suggests a strong association between Vanuatu’s trade liberalisation and the increased availability of the diverse range of imported products: fats and oils, meat and canned fish, processed dairy products, energy-dense beverages, and processed and packaged foods. While liberalisation and commitments under WTO trade agreements have changed the food availability and nutritional quality of Vanuatu’s food environment, there are several important and interesting caveats to note. The main points for discussion in this section focus on a series of liberalisation processes based on Vanuatu’s WTO commitments accompanying the changes in food imports and how these have contributed to shaping Vanuatu’s food environment by increasing less healthy food imports.

### Structural adjustment reforms: the use of tariffs and excise duties

As a WTO signatory in 2012, Vanuatu agreed to binding all its tariffs. While there has been an influx of less healthy food imports, the relationship is not straightforward. For some food categories, such as ice-cream and edible ices, savoury ready-to-eat snacks and sweet snacks, tariffs have remained unchanged, but the percentage import volumes of these foods has been increasing for a number of years. In the case of soft drink and cordial imports, despite a high applied tariff of 75% on soft drink imports and a 20% tariff on cordial imports remaining unchanged since 2008 at 20%, these imports still recorded an increase from 2016 to 2018. The additional excise duty and VAT applied to some of these food categories are likely to have contributed to the fluctuating trends in total food import volumes of these foods.

Many Pacific Island countries, including Vanuatu, have implemented excise taxes as a way to reduce consumer demand for unhealthy choices, but these need to be supported by other, complementary actions. In many instances, with the minimal increases in taxes and the ad hoc implementation of these, the intended impact on consumer behaviours has fallen short. Further to this, recent developments have also shown that the focus of excise tax increases has been on revenue generation rather than changing consumer behaviour and hardly any Pacific Island country (Vanuatu included) dedicates any of the excise tax revenue on targeted unhealthy products to the health sector [32].

### Vanuatu’s WTO commitments: WTO agreements on pre-shipment inspection, import prohibition and anti-dumping, countervailing or safeguard measures

To regulate the sale and availability of processed foods and sugar-sweetened beverages in Vanuatu’s food environment, various WTO agreements provide grounds for technical regulations to be prepared, adopted or applied by member states to protect human health and safety. Vanuatu has yet to maximise the general safeguards in line with these agreements and has only imposed import prohibitions on beef imports originating in Europe since the late 1990s for biosecurity reasons. This is in accordance with GATT Article XIII on Non-Discriminatory Administration of Quantitative Restrictions, Article XI, 2(c) on Quantitative Restrictions that allows for “import restrictions on any agricultural or fisheries product, imported in any form, necessary to the enforcement of governmental measures which operate” and pursuant to Vanuatu’s Customs (Prohibited Import) Regulations Order No.115 of 2014. Regarding the Agreement on Preshipment Inspection, Vanuatu does not carry out any preshipment inspection and has no laws, regulations or procedures and criteria in place to put this agreement into force. Moreover, Vanuatu has no intention of doing so as per their schedule of specific commitments. However, to ensure that the quality of goods shipped has complied with quality measures during the production process, it becomes important for countries to opt for pre-shipment inspection. Vanuatu should re-consider this commitment, as pre-shipment inspection can help reduce the risk of receiving poor-quality food products that are non-compliant with Vanuatu’s food related regulations.

To ensure fair trade and to protect against the dumping of goods and its trade distortive effects, the WTO Antidumping Agreement allows governments to act against dumping where it is hurting domestic industries or to cope with a sudden surge of foreign goods. While Vanuatu agreed to not applying any anti-dumping, countervailing or safeguard measures until it had implemented appropriate laws consistent with WTO agreements, it now hasdraft anti-dumping regulations under review. It must be cautioned that anti-dumping measures may pressurise the government to restrict the import of better and cheaper imports by calling them dumped commodities. Dumping should not be mistaken and simplified to mean cheap or low-priced imports. Rather it should only be taken up in its legal sense, that is, the export of goods lower than their normal value where the goods are low priced imports only in the relative sense – relative to their normal value.

### Vanuatu’s WTO commitments to foreign direct investment

The encouragement of foreign direct investment through Vanuatu’s commitments has also increased the availability of locally produced food preparations and processed foods and ultimately the consumption of these. As presented in Table 7, the production of foods for both local consumption and export has important implications for the nutritional quality of the food environment in Vanuatu. Food preparations such as bakery goods and biscuits, snack foods, soft drinks, chocolate products and other confectionary products are now produced locally. Further to this, Vanuatu’s commitment to have fewer restrictions on investment to promote local businesses has contributed to an increase in these food preparations via domestic industries as shown in Table 7 (see Additional file 4). These products are increasingly consumed as indicated in the 2017 Shop Survey [22] and the baseline study identifying households most at risk of poor nutrition outcomes in Vanuatu [23]. The rising availability of these less healthy products is most greatly associated with foreign direct investment and the progressive liberalisation of Vanuatu’s business environment with few restrictions on investment to promote domestic industries and small local businesses.

### Study limitations

The study was limited by gaps in available data – the segregation of data by countries exporting into Vanuatu, data relating to tariff rates, FDI investment and monetary data, and calculation of tariff-rate quotas for the identified focus foods. The limited availability of data made it difficult to demonstrate causality, nor could the importance of trade agreement provisions in driving change in nutrition quality and shaping Vanuatu’s food environment be effectively estimated.

## Conclusion

The analysis presented in this paper suggests that Vanuatu’s commitments to WTO Agreements do play an important role in shaping their food environment by increasing both healthy and less healthy imports. Strong associations between trade liberalisation and imported foods, especially ultra-processed foods were evident in the measured indicators. In terms of total food import volume, Vanuatu’s food trade with 32 WTO countries has contributed to the high levels of food import volumes. This has also seen a marked increase in ‘less healthy’ focus food imports namely fatty and other selected meat products, sugar, savoury snacks, ice-cream and edible ices and energy-dense beverages. Vanuatu’s binding tariffs has impacted import trends of ice-cream and edible ices, bakery products and confectionary. Although for some food categories such as crisps, snacks and noodles, a sharp increase in the importation of these is evident despite tariff rates remaining unchanged from 2008 to 2019. The encouragement of FDI through Vanuatu’s commitments has also increased the availability of locally produced food preparations, processed and ultra-processed foods through its 49 foreign-owned food related companies involved in food manufacturing and processing and the production of coffee, bakery products, confectionary, food preservatives, fish, local food products and meat, and the manufacturing, processing and packaging of palm oil, coconut oil, cooking oil, water, cordial juice, flavoured juices, soft drinks and alcoholic beverages. These were largely produced for local consumption. It has also seen the substantive engagement of 32 domestic industries in food and beverage production.

An assessment of WTO provisions relating to domestic policy space and governance in Vanuatu shows that the current legal and regulatory environment for food in Vanuatu remains fragmented. Trade agreements are increasingly including commitments, usually aimed at harmonising national measures affecting traded goods and this potentially constrains domestic policy making [33]. While major policy changes are needed to create environments that encourage healthy food consumption [17], the government of Vanuatu remains challenged in this area, as policy processes continue to lack cohesion across multiple sectors and the prioritisation of economic and trade interests above public health [34]. However, there are many principles within Vanuatu’s commitments under the WTO agreements that provide opportunity for Vanuatu to enhance policy coherence between trade and health and to strengthen its nutrition policy space. To minimise the risk of incurring challenges under WTO agreements, public health actors and policymakers must understand the accession context and specific clauses of the WTO which can impact policy and ultimately the scope and scale of food production and consumption.

## Supplementary Information

**Additional file 1.** Tariff rates for selected less healthy focus foods.

**Additional file 2.** Tariff rates for selected healthy focus foods

**Additional file 3.** Transnational corporations engaged in the food and beverage sector operating in Vanuatu in 2019.

**Additional file 4.** Domestic industries engaged in the food and beverage sector operating in Vanuatu in 2019.
