# Supplementary material for: Analysing the impact of trade agreements on national food environments: the case of Vanuatu
Source: Global Health. 2021 Sep 16;17:107. doi: 10.1186/s12992-021-00748-7 (PMC8447725; doi:10.1186/s12992-021-00748-7)
Supplement: Supplementary file 1 — Additional file 1. Tariff rates for selected less healthy focus foods. [file 12992_2021_748_MOESM1_ESM.pdf]

Additional File 1: Tariff rates for selected less healthy focus foods

Table 4: Tariff rates for less healthy focus foods

**Less healthy Focus Food**	**2008**	**2009**	**2010**	**2011**	**2012**		**2013**		**2014**		**2015**		**2016**	
	Applied	Applied	Applied	Applied	Applied	Bound	Applied	Bound	Applied	Bound	Applied	Bound	Applied	Bound
Palm Oil	10%	10%	10%	10%	10%	40%	10%	40%	10%	40%	10%	40%	10%	40%
Corn Oil	10%	10%	10%	10%	10%	40%	10%	40%	10%	40%	10%	40%	10%	40%
Hydrogenated fats, lard, dripping, TINCOL etc.	10%	10%	10%	10%	10%	40%	10%	40%	10%	40%	10%	40%	10%	40%
Margarine	10%	10%	10%	10%	10%	30%	10%	30%	10%	30%	10%	30%	10%	30%
Butter	15%	15%	15%	15%	15%	30%	15%	30%	15%	30%	15%	30%	15%	30%
Peanut Butter	20%	20%	20%	20%	10%		10%		10%		10%		10%	
Sausage	20%	20%	20%	20%	20%	40%	20%	40%	20%	40%	20%	40%	20%	40%
Ham, bacon, salami, jerky, cold cuts, chicken nuggets	20%<br>Chicken: 30%	20%<br>Chicken: 30%	20%<br>Chicken: 30%	20%<br>Chicken: 30%	30%<br>Chicken: 30%	40%<br>Chicken: 55%	30%<br>Chicken: 30%	40%<br>Chicken: 55%	30%<br>Chicken: 30%	40%<br>Chicken: 55%	30%<br>Chicken: 30%	40%<br>Chicken: 55%	30%<br>Chicken: 30%	40%<br>Chicken: 55%
Canned Fish	20%<br>Sardine: 20%	20%<br>Sardine: 20%	20%<br>Sardine: 20%	20%<br>Sardine: 30%	20%<br>Sardine: 30%	40%<br>Sardine: 30%	20%<br>Sardine: 30%	20%<br>Sardine: 30%	20%<br>Sardine: 30%	20%<br>Sardine: 30%	20%<br>Sardine: 30%	20%<br>Sardine: 30%	20%<br>Sardine: 30%	20%<br>Sardine: 30%
Canned Meat	20%	20%	20%	20%	20%	40%	20%	40%	20%	40%	20%	40%	20%	40%
Processed Cheese	15%	15%	15%	15%	15%	30%	15%	30%	15%	30%	15%	30%	15%	30%
Fruit based/flavoured yoghurt	30%	30%	30%	30%	15%	40%	15%	40%	15%	40%	15%	40%	15%	40%
Ice-cream and edible ices	30%	30%	30%	30%	30%	65%	30%	65%	30%	65%	30%	65%	30%	65%
Cordial/Juices	20%	20%	20%	20%	20%	65%	20%	65%	20%	65%	20%	65%	20%	65%
Soft drink	75%	75%	75%	75%	75%	75%	75%	75%	75%	75%	75%	75%	75%	75%
Electrolyte/Sports drinks	75%	75%	75%	75%	75%	75%	75%	75%	75%	75%	75%	75%	75%	75%
Sugar & other caloric swt.	10%	10%	10%	10%	10%	40%	10%	40%	10%	40%	10%	40%	10%	40%
Crips & snacks	20%	20%	20%	20%	20%	40%	20%	40%	20%	40%	20%	40%	20%	40%
Noodles	10%	10%	10%	10%	10%	40%	10%	40%	10%	40%	10%	40%	10%	40%
Confectionary	10%	10%	10%	10%	10%	40%	10%	40%	10%	40%	10%	40%	10%	40%
Sweet Biscuits	10%	10%	10%	10%	10%	40%	10%	40%	10%	40%	10%	40%	10%	40%

<u>Cont'd: Table 2</u>

**Less healthy Focus Food**	**2017**		**2018**		**2019**	
	Applied	Bound	Applied	Bound	Applied	Bound
Palm Oil	10%	40%	10%	40%	10%	40%
Corn Oil	10%	40%	10%	40%	10%	40%
Hydrogenated fats, lard, dripping, TINCOL etc.	10%	40%	10%	40%	10%	40%
Margarine	0%	30%	0%	30%	0%	30%
Butter	15%	30%	15%	30%	15%	30%
Peanut Butter	20%		20%		20%	
Sausage	20%	40%	20%	40%	20%	40%
Ham, bacon, salami, jerky, cold cuts, chicken nuggets	30%<br>Chicken: 30%	40%<br>Chicken: 55%	30%<br>Chicken: 30%	40%<br>Chicken: 55%	30%<br>Chicken: 30%	40%<br>Chicken: 55%
Canned Fish	20%<br>Sardine: 30%	40%<br>Sardine: 30%	20%<br>Sardine: 30%	20%<br>Sardine: 30%	20%<br>Sardine: 30%	20%<br>Sardine: 30%
Canned Meat	20%	40%	20%	40%	20%	40%
Processed Cheese	15%	30%	15%	30%	15%	30%
Fruit based/flavoured yoghurt	15%	40%	15%	40%	15%	40%
Ice-cream and edible ices	30%	65%	30%	65%	30%	65%
Cordial/Juices	20%	65%	20%	65%	20%	65%
Soft drink	75%	75%	75%	75%	75%	75%
Electrolyte/Sports drinks	75%	75%	75%	75%	75%	75%
Sugar & other caloric sweeteners	10%	40%	10%	40%	10%	40%
Crips & snacks	20%	40%	20%	40%	20%	40%
Noodles	10%	40%	10%	40%	10%	40%
Confectionary	10%	40%	10%	40%	10%	40%
Sweet Biscuits	10%	40%	10%	40%	10%	40%
