# Supplementary material for: Analysing the impact of trade agreements on national food environments: the case of Vanuatu
Source: Global Health. 2021 Sep 16;17:107. doi: 10.1186/s12992-021-00748-7 (PMC8447725; doi:10.1186/s12992-021-00748-7)
Supplement: Supplementary file 2 — Additional file 2. Tariff rates for selected healthy focus foods [file 12992_2021_748_MOESM2_ESM.pdf]

Additional file 2: Tariff rates for selected healthy focus foods

Table 5: Tariff rates for healthy focus foods

**Healthy Focus Food**	**2008**	**2009**	**2010**	**2011**	**2012**	**2013**	**2014**	**2015**	**2016**	**2017**	**2018**	**2019**
	Applied	Applied	Applied	Applied	Applied	Applied	Applied	Applied	Applied	Applied	Applied	Applied
Citrus	30%	30%	30%	30%	30%	30%	30%	30%	30%	30%	30%	30%
Fresh apples	30%	30%	30%	30%	30%	30%	30%	30%	30%	15%	15%	15%
Fresh grapes	30%	30%	30%	30%	30%	30%	30%	30%	30%	15%	15%	15%
Fresh tomatoes	30%	30%	30%	30%	30%	30%	30%	30%	30%	30%	30%	30%
Garlic	30%	30%	30%	30%	30%	30%	30%	30%	30%	5%	5%	5%
Onions	30%	30%	30%	30%	30%	30%	30%	30%	30%	5%	5%	5%
Leeks and other alliaceous vegetables	30%	30%	30%	30%	30%	30%	30%	30%	30%	30%	30%	30%
Cauliflowers and broccoli	30%	30%	30%	30%	30%	30%	30%	30%	30%	30%	30%	30%
Other cabbages and cauliflowers	30%	30%	30%	30%	30%	30%	30%	30%	30%	30%	30%	30%
Cabbage lettuce	30%	30%	30%	30%	30%	30%	30%	30%	30%	30%	30%	30%
Other lettuce, other vegetables	30%	30%	30%	30%	30%	30%	30%	30%	30%	30%	30%	30%
Carrots and turnips	30%	30%	30%	30%	30%	30%	30%	30%	30%	30%	30%	30%
Potatoes	30%	30%	30%	30%	30%	30%	30%	30%	30%	30%	30%	30%
Celery	30%	30%	30%	30%	30%	30%	30%	30%	30%	30%	30%	30%
Dried leguminous vegetables, split lentils, chickpeas and kidney beans	30%	30%	30%	30%	30%	30%	30%	30%	30%	15%	15%	15%
Rice (brown or white)	15%	15%	15%	15%	15%	15%	15%	15%	15%	15%	15%	15%
Rolled Oats or Oatmeal	5%	5%	5%	5%	5%	5%	5%	5%	5%	5%	5%	5%
Healthy breakfast cereals	10%	10%	10%	10%	10%	10%	10%	10%	10%	10%	10%	10%

N.B. Bound tariff data for each food category was not provided so applied tariff rates only are reflected in this table.
