# Supplementary material for: Analysing the impact of trade agreements on national food environments: the case of Vanuatu
Source: Global Health. 2021 Sep 16;17:107. doi: 10.1186/s12992-021-00748-7 (PMC8447725; doi:10.1186/s12992-021-00748-7)
Supplement: Supplementary file 3 — Additional file 3. Transnational corporations engaged in the food and beverage sector operating in Vanuatu in 2019. [file 12992_2021_748_MOESM3_ESM.pdf]

Additional file 3: Transnational corporations engaged in the food and beverage sector operating in Vanuatu in 2019

Table 6: Transnational corporations engaged in the food and beverage sector operating in Vanuatu in 2019
*F&B – Food & Beverage Service
*N/A – Not Available

Parent Country	TFC	Type of business	Trade Activity	Year of investment	Total actual investment (MVT)
Japan	Santo Meat Packers Limited	Abattoir and Meat Packing	F&B	1999	0
France	Vanuatu Beverage Ltd	Manufacturing of Drinks, Retail of finished Products, produced at the beverage factory, Wholesale and retail of beverages	F&B	1998	894,000,000
JV Local	Wong Bakery	Bakery	F&B	2000	110,000,000
Australia	Tanna Coffee Development Company Ltd	Coffee Processing, Packaging, Wholesale/Retail, Management Services & Consultants (Consultancy for Agriculture Development and Associated Services)	F&B	2001	190,784,000
Japan	Fuji Company Limited	Food Processing, Restaurant, Bar	F&B	2002	24,000,000
Japan	Fuji Company Limited	Food Processing, Restaurant, Bar	F&B	2002	24,000,000
New Zealand	Daming Store	Bakery and Confectionaries, Import and Re-Export, Retail and Wholesale (Main Shop), Retail and Wholesale (Branch), Butchery, Restaurant	F&B Imports Re-exports	2004	300,000,000
Australia	Vanuatu Virgin Coconut Oil Ltd	Coconut Processing, Import, Retail & Export Exempted	Imports	2004	391,000,000
China	Vanuatu Palm Oil Limited	Oil Processing, Packaging, Agriculture, Export Exempted	F&B Export	2005	53,000,000
China	China Cake Shop	Production of Cake, Retail	F&B	2005	70,000,000
Australia	Azure Pure Water Limited	Water Distillation, Production & Bottling of Juice, Wholesale/Retail of water and Soft Drinks	F&B	2006	177,000,000
Australia	Pechans Continental Small Goods	Production of Meat loaf, sausages & small goods, Wholesale/Retail of above goods only	F&B	2007	7,500,000
France	Vanuatu Organics Limited	Manufacturing & Processing of Agriculture Products, Retail of Processing Items, Storage & Processing items,	Agricultural Processing	2007	0
		Agriculture Farming, Management Services & Consultants (Agriculture, Fisheries, production, processing, value addition & marketing) Export of Finished Products-Exempted			
United Kingdom	Nuts N' Oils Vanuatu	Manufacturing of oil, Production of Stockfeed, Wholesale/Retail of Oil, Management Services	Retail Production	2008	15,000,000
France	Vanuatu Brewing Limited	Manufacturing, Retailer, Retail of Beers, Beverage & Liquor, Bar, Café, Restaurant	F&B	2008	307,700,000
JV Other	Victoria Café	Bakery,Import, Wholesale/Retail, Restaurant, Café, Bar	F&B	2008	3,500,000
China	SinoVan Fisheries Limited	Fish processing, Imports, Wholesale/Retail, Fishing Export-Exempt	F&B Imports	2008	10,380,000
Australia	Coconut Oil Production Santo Limited	Production of Coconut Oil Mill and Export of Coconut Oil and Copra Meal	Exports	2009	522,092,566
Australia	Nambawan Brewing Company Limited	Breweries, Distilleries, Retail/wholesale	F&B	2010	17,000,000
China	Vanuatu Noni Life Technology Limited	Distilleries (Soap Cleaning, preparation/beverages), Business that import and Re-Export goods without transforming them, Wholesale and Retail of noni products (specialty shop) Export-Exempted	F&B Imports Re-exports	2011	165,000,000
Australia	South Pacific Botanicals Limited	Manufacturing of Oil and Other Related Products	Production	2011	2,435,793
China	South Pacific Agriculture Group Limited	Manufacture of seafood products, farming of seafood, Export	Exports F&B	2011	0
France	Vanuatu International Food Market	Manufacture of Food Products and Export	F&B Exports	2011	2,500,000
Australia	Operations Limited (Pure Spirit Limited)	Distilleries	F&B	2011	5,624,838
JV Local	Global Fresh Limited	Meat Production, Commercial Construction	F&B Construction	2012	13,318,472
Australia	Pacific Provender Limited	Distilleries, Oil Mill, Manufacture of Perfume and Food Oil, Agriculture Organic Consultant, Planters	F&B Agricultural Processing	2013	20,200,000
New Zealand	Pacific Aluminium Fabrications and Glass Limited	Bakery, Restaurants, Café, Bar	F&B	2013	60,100,000
Israel	Attar Café And Bakery	Bakery, Restaurants, Café,	F&B	2013	27,500,000
New Zealand	Tanna Fams	Manufacture of Food Products (Tafea), Oil Mill, Agriculture (Tafea) ,Manufacture of Soap and Cleaning Preparations (Port Vila), Manufacture of Coconut Oil (Port Vila), Export	F&B Agricultural Production	2014	20,600,000
			and Processing Exports		
France	Le Fournil De Vila	Bakeries and Confectioneries, Restaurants	F&B	2014	28,950,000
China	Nature Company Limited	Manufacturing and Bottling of Liquor and Spirits, Manufacture of Noni Juice, Retail & Wholesale of Metal Pattern Gates, Beds, Fencing, Bolt, Building Frames	F&B Construction	2014	106,000,000
France	Vanuatu Delicious Paris	Manufacturing of Food Products and Preservation and Tour Agent	F&B	2014	7,000,000
Australia	Torba Trading Co Limited	Production of Coconut Oil, Export	Exports	2014	9,000,000
France	Aelan chocolate Makers Limited	Manufacture of Chocolate Products	F&B	2014	3,000,000
Australia	Plantation Paradise Limited	Processing of Coconut Oil, Copra Plantation, Cocoa Plantation, Cattle Farmers	Agricultural Processing	2014	45,000,000
France	Agri - Santo	Manufacture of coconut oil, Export	Exports	2014	4,000,000
China	Brother	Manufacture of Food Products, Retail, Restaurant, Café, Take- Away	F&B	2014	60,000,000
China	Golden Palm	Manufacture of food products, Bakeries, Confectioneries, Manufacture of all beverage excluding alcoholic drinks, Import, Hardware, Retail of vehicles spare parts, Holiday homes, Cafe, Commercial Building Constructions	F&B Construction	2014	0
Australia	Aore Island Coffee	Processing Cocoa, coffee and coconut oil, Modern Bungalows, Restaurant, Café, Coconut and Coffee Plantation	F&B	2015	42,000,000
British	Pacific Rim (Vanuatu) Limited	Oil Mill, Manufacture of Coconut Water, Manufacture of Animal Feed, Export	Agricultural Production and Processing	2015	16,200,000
JV Other	The Distillery Company Limited	Distillery of rum and liquor, Wholesale and retail of liquor - Export	F&B	2016	25,100,000
China	Sky Rock International Investment Limited	Bakeries and Confectioneries, Wholesale and retail, Boutique (specialty shop), Hotel, Restaurant, Bar, Cattle farming	F&B	2017	N/A
China	Pacific Islands Economic Developments	Palm oil mill, Sawmill, Coconut shell carbon productions and Wholesale and retails services	Agricultural Production and Processing	2017	N/A
JV Local	Paradise Spring Water Limited	Manufacture of plastic water bottle and manufacturing of natural spring water & other non - alcohol beverages	F&B	2017	N/A
JV Other	Island Distillery Limited	Manufacture of local food products, Ship builders and repairs, Manufacture of oil milk, Business that import and re-export goods without transforming them, Poultry farming and Piggery farming	F&B Construction Agricultural Processing	2017	N/A
Joint Venture Local	Diam Thomas Trading Co Limited	Processing of powder kava for export, Export of powder kava	Exports	2017	17,270,000
Joint Venture Other	Pacific Pride Limited	Copra production, Training and upskilling local copra producers to produce quality copra - Copra export and Local purchase of copra	Agricultural capacity building	2018	N/A
New Zealand	Evco Pacific Vanuatu Ltd	Coconut Oil Mill, Manufacture of Food Product	F&B Agriculture	2018	N/A
Joint Venture Local	Shefa Bakery Limited	Bakery, Café, Take away services	F&B	2018	0
