# Supplementary material for: Analysing the impact of trade agreements on national food environments: the case of Vanuatu
Source: Global Health. 2021 Sep 16;17:107. doi: 10.1186/s12992-021-00748-7 (PMC8447725; doi:10.1186/s12992-021-00748-7)
Supplement: Supplementary file 4 — Additional file 4. Domestic industries engaged in the food and beverage sector operating in Vanuatu in 2019. [file 12992_2021_748_MOESM4_ESM.pdf]

Additional file 4: Domestic industries engaged in the food and beverage sector operating in Vanuatu in 2019

Table 7: Domestic industries engaged in the food and beverage sector operating in Vanuatu in 2019
*N/A – Not Available

Business Name	Products	Year of registration	Market	Location
Switi	Ice cream, Yoghurts	2015	100% domestic	Urban
Cake Fantasy	Celebration cakes and associated goods	2015	100% domestic	Urban
ACTIV	Chocolate, chocolate nib, cocoa powder, cocoa butter	2014	80% domestic, 20% export	Urban
Boucheri Traverso	Meat	2014		Urban
Azure Pure Water	Bottled water	2015	100% domestic	Urban
Chiko Farm Products Ltd	Chicken meat, egg products, chicken feed production	2015	100% domestic	Rural
Vanuatu Beverage	Orchy (orange, mango, passionapple, pine orange), cordials, prima cordial, mix cordial, Vanuatu water, alcohol, slushy with 8% alcohol, imported liquors	2015	95% domestic 5% export	Urban
Nambawan Brewing Company Ltd	Beer, cider, spirits, soft drinks, alcoholic drinks, water	2016	99% domestic 1% export	Urban
Vanuatu Direct	Honey, island foods, spicy carnibal sauce, health shot papaya and kava, dried fruits and nuts, nut butters, fruit juices, frozen delights, processed meat items	2014	20% domestic 80% export	Rural
Vanuatu Abattoirs Ltd	Chilled and frozen boneless beef, beef by-products	2015	30% domestic 70% export	Rural
At Your Service Catering	Meat pies, sausage rolls	2015	100% domestic	N/A
Pechans Continental Small Goods	Sausages, smoked fish, pate, smoked beef, smoked chicken	N/A	100% domestic	N/A
Pepe's Farm	Fresh poultry	N/A	100% domestic	N/A
T/Mel Butchery (Tina's)	Sausages, bacon, fresh meat product	2015	100% domestic	Urban
South Pacific Meat Supplies Ltd	Meat products	2015	100% domestic	N/A
Pure Spirits Ltd	Spirits, Liquors	2015	100% domestic	Urban
Vanuatu Brewing Ltd	Tusker beer, Vanuatu Bitter,	2015	99% domestic 1% export	Urban
Elcress Agra Products Ltd	Meat, cooking oil	2015	100% domestic	N/A
Kandy's Kitchen Creations	Chocolate sauce, chai tea syrup, tomato sauce, pawpaw chutney, cucumber pickle, fruit paste, muesli, mango chutney, chill and garlic, chill jam and chilli paste	2015	100% domestic	N/A
F.P.F. Company Limited trading as Tanna Water	Mineral water, food products, beverage	2015	50% domestic 50% export	N/A
Wong Sze Sing	Corned beef, canned curry beef, canned curry chicken, beef & taro, chicken & taro	2015	100% domestic	Rural (Santo)
Vanuatu's Own	Red Bull's vodka, Karl's fine whiskey, Sanny Bel top dry gin	2016	N/A	Rural
Lapita Cafe	Food processing, taro chips, banana chips, manioc chips, manioc flour, chutney, chilli tomato sauce, choko relish	2015	N/A	Urban
Mahesh Oil Industries	Processing peanut oil		Export	Rural (Santo)
Eco Fresh Ltd	Jam	2017	N/A	N/A
CL CHEMSULT	Local fruit wines, distilled spirits,	2018	N/A	N/A
Tanna Island Direct Trade Coffee Exporters	Dry green bean coffee	2018	Domestic and export	N/A
Bruns Services	Frozen taro, frozen manioc, frozen yam, frozen coco yam, manioc leaf, taro leaf, choco bean	2019	Export	N/A
Sino-Van Fisheries	Frozen tuna, frozen bycatch	2019	Domestic and export	N/A
French Bakery Limited	Bakery, Pastry, Take away services	2019	Domestic	N/A
Global Organic Products Distributor	Dried spices, dry vegetables, dry fruits	2019	Domestic and export	N/A
Vanuatu Organic FOOD Limited	Manioc Flour	2019	Export	N/A
